# Comments on “Adherence to oral zinc supplementation in the management of acute diarrhoeal disease among under-5 children: a systematic review and meta-analysis”

**DOI:** 10.1017/S0950268826101447

**Published:** 2026-04-17

**Authors:** Sushma Narsing Katkuri, Varshini Vadhithala, Arun Kumar, Sushma Verma, Dhanya Dedeepya

**Affiliations:** 1 Department of Community Medicine, Malla Reddy Instiute of Medical Sciences, Malla Reddy Vishwavidyapeeth, Suraram, Hyderabad 500055, Telangana, India; 2 Dr. D. Y. Patil Medical College, Hospital and Research Centre, Dr. D. Y. Patil Vidyapeeth (Deemed-to-be-University), Pimpri, Pune 411018, Maharashtra, India; 3 Faculty of Pharmaceutical Sciences, Graphic Era Hill University, Dehradun, India; 4 Centre for Promotion of Research, Graphic Era Deemed University, Dehradun, India; 5 Department of Pharmaceutics, Noida Institute of Engineering & Technology (Pharmacy Institute) Plot No.19, Knowledge Park-II, Greater Noida, India; 6 Saveetha Medical College and Hospital, Saveetha Institute of Medical and Technical Sciences, Saveetha University, Chennai, India; 7 Department of Anatomy, School of Medical Sciences and Research, Sharda University, Greater Noida, India

Dear editor,

We read with interest the systematic review and meta-analysis on adherence to oral zinc supplementation for acute diarrhoeal disease in children under five [1]. The topic is important for translating guideline recommendations into real-world practice, and the authors should be commended for focusing on adherence rather than efficacy alone. Their prospective registration, broad search strategy, and explicit use of a structured risk of bias tool are notable strengths that align with contemporary guidance for systematic reviews [2].

Several methodological issues, however, warrant consideration when interpreting the pooled adherence estimates. First, although the review is framed as including observational studies, the eligibility criteria and final sample also incorporate at least one prospective open-label interventional study. Mixing observational and interventional designs is not inherently problematic, yet it should be clearly acknowledged and justified, since different designs may have distinct patterns of adherence, measurement error, and risk of bias [3].

Second, the application of the NHLBI tool for observational cohort and cross-sectional studies is described as yielding a 0–6 summary score, with higher scores interpreted as a higher risk of bias. The original developers caution against creating simple summary scores and against reversing the intuitive direction of such scores because different items are not psychometrically equivalent and do not contribute equally to bias [4]. Without specifying which items were selected, how they were coded, and why higher values denote worse quality, readers may struggle to understand how study-level bias influenced the synthesis. This is particularly relevant because one included study is interventional, for which an observational tool may not be fully appropriate.

Third, the meta-analytic pooling of adherence proportions occurs in the presence of extremely high between-study heterogeneity (I^2^ values close to 100%) and clear evidence of publication bias. When outcome definitions, settings, and measurement methods differ substantially across studies, a single pooled prevalence may have limited meaning [3]. A stronger approach would be to emphasise the range and pattern of adherence across contexts, supplemented by exploratory subgroup or sensitivity analyses based on setting, regimen duration, and adherence definition, rather than relying on a precise summary estimate.

Finally, the review treats adherence as a homogeneous construct, although the included studies define it variably as completion of a 10- or 14-day course, or as achieving a minimum number of days based on caregiver report. Methodological literature on outcome harmonisation stresses that conceptually distinct definitions should be handled with caution in quantitative synthesis, or at least subjected to careful stratification [2, 3]. Explicitly mapping and grouping these definitions would help readers understand whether low adherence reflects shorter duration, missed doses, early discontinuation after symptom resolution, or other behavioural patterns.

While this review addresses a clinically important question and is methodologically transparent in several respects, the mixing of designs, non-standard risk of bias scoring, very high heterogeneity, and variable outcome definitions suggest that the pooled adherence estimates should be interpreted cautiously. We hope these comments assist readers and editors in appraising the evidence and inform future work on adherence to zinc and other supportive therapies in childhood diarrhoea.

## Data Availability

Not applicable.

## References

[1] Pradhan SK, et al. (2025) Adherence to oral zinc supplementation in the management of acute diarrhoeal disease among under-5 children: A systematic review and meta-analysis. Epidemiology and Infection 153, e129. 41178278 10.1017/S0950268825100733 PMC12641308

[2] Page MJ, et al. (2021) The PRISMA 2020 statement: An updated guideline for reporting systematic reviews. BMJ 372, n71. 33782057 10.1136/bmj.n71 PMC8005924

[3] Higgins JPT, et al. (ed.). (2024) Cochrane Handbook for Systematic Reviews of Interventions. version 6.5 (updated August 2024). Cochrane, www.cochrane.org/handbook. (10 November 2025).

[4] Sanderson S, Tatt ID and Higgins JP (2007) Tools for assessing quality and susceptibility to bias in observational studies in epidemiology: A systematic review and annotated bibliography. International Journal of Epidemiology 36(3), 666–676. 17470488 10.1093/ije/dym018

